# Probiotics as antioxidant, antistress, and growth-enhancing agents in monogastric animals: a narrative review

**DOI:** 10.3389/fvets.2026.1815504

**Published:** 2026-04-21

**Authors:** Victory Osirimade Sumanu, Kennedy Ejebhiare Osuidia, Ifeanyichukwu Chukwuemeka Egbuniwe, Vinny Naidoo, Marinda Oosthuizen, Joseph Panashe Chamunorwa, Lyndy Joy McGaw

**Affiliations:** 1 Department of Paraclinical Sciences, Faculty of Veterinary Science, University of Pretoria, Onderstepoort, South Africa; 2 Department of Veterinary Physiology and Biochemistry, Faculty of Veterinary Medicine, University of Benin, Benin City, Nigeria; 3 Department of Veterinary Tropical Diseases, Faculty of Veterinary Science, University of Pretoria, Onderstepoort, South Africa; 4 Department of Anatomy and Physiology, Faculty of Veterinary Science, University of Pretoria, Onderstepoort, South Africa

**Keywords:** animal nutrition, feed additives, gut health, monogastric animals, probiotics

## Abstract

Probiotics, defined as living microorganisms that confer health benefits when administered in adequate amounts, have demonstrated significant potential in enhancing productivity and physiological resilience in monogastric animals, including poultry, companion animals, and swine. This narrative review synthesizes recent evidence on the antioxidant, anti-inflammatory, antistress, and growth-promoting functions of commonly studied probiotic genera, such as *Lactobacillus*, *Bifidobacterium*, *Bacillus*, and *Saccharomyces*. Probiotics improve physiological homeostasis through multiple mechanisms. As antioxidants, they enhance endogenous enzyme activities including catalase, superoxide dismutase, and glutathione peroxidase resulting in 15–40% increases in enzyme activity and 20–45% reductions in lipid peroxidation markers, indicating improved oxidative balance. Antistress effects are mediated via modulation of the gut–brain axis, lowered corticosterone levels, and immune regulation through cytokine changes (IL-6, TNF-α, IL-10). Additionally, probiotics support intestinal integrity, reduce pathogenic load, and enhance nutrient utilization, contributing to 5–12% improvements in feed efficiency under controlled conditions. The efficacy of probiotics depends on strain specificity, dosage, delivery method, and host factors, emphasizing the need for precise formulation. In conclusion, probiotics offer a promising nutritional strategy for improving health, stress tolerance, and productivity in monogastric species. Future research should focus on elucidating molecular mechanisms, including Nrf2 signalling and gut barrier regulation, optimizing dosing strategies and exploring synergistic combinations with prebiotics and phytogenic additives. Such targeted approaches can maximize the benefits of probiotics and support sustainable, precision-driven monogastric production systems.

## Highlights

- Probiotics influence the antioxidant defense systems in monogastric animals.
- The supplementation of probiotics improves gut integrity, immune competence, and microbial balance in monogastric species.
- Dietary probiotics mitigates physiological and oxidative stress.
- Probiotics provide alternatives to antibiotic growth promoters in monogastric animal production systems.
- Feed efficiency and growth performance are enhanced through improved nutrient digestibility and metabolic efficiency.

## 1 Introduction

Probiotics are living microorganisms, typically bacteria or yeast, which, when administered in adequate amounts, confer measurable health benefits on the host (1–4). The term “probiotic” originates from the Greek words *pro* (“for”) and *biota* (“life”), reflecting their role in supporting beneficial biological functions (5). Documented benefits of probiotics include improved gastrointestinal health, enhanced immune responses, and reduced susceptibility to specific diseases (6). Importantly, these effects are either genus-, species- or strain-specific, meaning that they produce distinct biological outcomes and should be selected according to the targeted physiological benefits (7, 8). Probiotics are generally considered non-pathogenic in healthy hosts (9) and are often derived from organisms that naturally form part of the host microbiota (10). Although they do not typically establish permanent colonization in the gastrointestinal tract, probiotics can modulate the resident microbial community, influencing its composition, metabolic activity, and overall functionality during their transient presence (11, 12). To achieve their intended effects, microbial viability must be preserved during processing, storage, and administration (115), as environmental factors such as temperature, pH, and moisture can impact their stability and functional performance (13, 14).

Probiotic applications are well-documented across a wide range of monogastric species, including poultry (chickens, turkeys, ducks, quail), swine, rabbits, and companion animals such as dogs, cats and even horses (8, 15–19). In these species, probiotics improve gut health, feed efficiency, growth performance, immune function, and stress stability. Regulatory frameworks for probiotics vary by country, and product claims are subject to oversight to ensure scientific validation (20–22). Beyond gastrointestinal effects, research is expanding to explore probiotics’ roles in mental health, metabolic regulation, dermatological conditions, and systemic inflammatory disorders (5, 23–25). The objective of this article is to provide a comprehensive review of the mechanisms, health benefits, and growth-promoting effects of probiotics in monogastric farm and companion animals, highlighting their antioxidant, anti-stress, and immunomodulatory functions, and to discuss strategies for optimized, species-specific application.

## 2 Mechanisms of action of probiotics in the gastrointestinal tract

Probiotics exert their beneficial effects in the gastrointestinal (GI) tract through multiple mechanisms that promote intestinal balance and overall health. By shaping gut microbiota composition, supporting mucosal barrier integrity, and modulating host physiological responses, probiotics enhance gut function, nutrient utilization, immune competence, and general wellbeing.

### 2.1 Competitive exclusion

Probiotics inhibit the growth and colonization of pathogenic microorganisms primarily through competitive exclusion, competing for nutrients and adhesion sites on the intestinal epithelium. By adhering to the gut mucosa, probiotics block pathogens from attaching, reducing infection risk and limiting harmful bacterial establishment. They may also produce antimicrobial metabolites, such as organic acids and bacteriocins, which further suppress pathogen proliferation. Strains such as *Saccharomyces boulardii*, *Lacticaseibacillus rhamnosus*, and *Bifidobacterium bifidum* effectively occupy gut niches that might otherwise be colonized by pathogens, supporting microbial balance and intestinal health (26–28).

### 2.2 Production of antimicrobial substances

Many probiotic microorganisms produce a variety of antimicrobial substances that inhibit pathogenic bacteria and promote gut health (29, 30). Key mechanisms include the production of organic acids, such as lactic acid and acetic acid, which lower the gastrointestinal pH and create an unfavorable environment for the proliferation of harmful microbes (29). In addition, probiotics synthesize antimicrobial peptides, notably bacteriocins, which target pathogenic bacteria by disrupting cell membrane integrity, inhibiting cell wall synthesis, or interfering with essential metabolic pathways (29). Specific strains demonstrate well-characterized antimicrobial activities; for instance, *Lacticaseibacillus* species produce lactic acid, while *Lacticaseibacillus reuteri* secretes reuterin, a broad-spectrum antimicrobial compound effective against Gram-positive and Gram-negative pathogens (29, 31).

*In vivo* studies have confirmed these effects, showing that probiotic supplementation can significantly reduce intestinal colonization by pathogens such as *Escherichia coli, Salmonella* spp., and *Clostridium perfringens* in poultry and swine, thereby decreasing the incidence of gastrointestinal infections and associated inflammatory responses (29). Perez-Guerra et al. (29) also reported that the administration of probiotics to piglets via feed improved their general performance and health status. Moreover, antimicrobial activity often works synergistically with host immune modulation: probiotics can enhance mucosal immunity by stimulating the production of secretory IgA, promoting the activity of macrophages and dendritic cells, and modulating cytokine profiles, which collectively improve the host’s ability to resist infection (29, 31). Through these complementary actions direct pathogen inhibition, modulation of gut environment, and reinforcement of host immune defences probiotics help maintain microbial balance, protect intestinal barrier integrity, and support overall gastrointestinal health (17, 32).

### 2.3 Modulation of the immune system

Certain probiotic genera, including *Bifidobacterium* and *Saccharomyces*, can significantly influence the host’s immune system under specific conditions, promoting a balanced and effective immune response (33). These immunomodulatory effects are mediated through multiple mechanisms, such as the stimulation of immune cell activity, production of bioactive metabolites, and reinforcement of gut barrier integrity (33). Probiotics interact with immune cells within the gut-associated lymphoid tissue (GALT), including macrophages, dendritic cells, and T lymphocytes, thereby shaping both local and systemic immunity (34, 74).

Probiotics modulate cytokines by interacting with epithelial receptors and gut immune cells, thereby downregulating pro-inflammatory cytokines such as IL-6 and TNF-α while upregulating anti-inflammatory cytokines such as TGF-β and IL-10 to enhance immune balance (34, 74). By modulating cytokine profiles, probiotics promote the secretion of anti-inflammatory cytokines (e.g., IL-10 and TGF-β) while suppressing pro-inflammatory cytokines (e.g., TNF-α, IL-6), contributing to immune homeostasis and the prevention of excessive inflammatory responses (34, 74). Furthermore, *Bifidobacterium* and *Saccharomyces* enhance mucosal immunity by stimulating secretory immunoglobulin A (sIgA) production, strengthening the gut’s first line of defense against pathogens (2, 33). These effects are not limited to local gut immunity; probiotics can also modulate systemic immune responses, influencing distant organs and overall host health. Collectively, the immunomodulatory properties of these probiotics highlight their potential as natural adjuvants for improving disease resistance and maintaining immune balance in monogastric animals.

### 2.4 Enhancement of gut barrier function

Probiotic species such as *Saccharomyces* and *Lacticaseibacillus* play a critical role in maintaining and reinforcing the integrity of the intestinal barrier, which is essential for preventing the translocation of harmful substances, toxins, and pathogenic microorganisms from the gut lumen into systemic circulation (31). These probiotics enhance barrier function by strengthening tight junctions between intestinal epithelial cells, thereby reducing intestinal permeability and limiting potential inflammatory responses. They achieve this by enhancing mucus production, strengthening tight junction proteins, lowering inflammation, and producing metabolites like short-chain fatty acids that influence epithelial repair and barrier integrity (31). In addition, they stimulate the production and secretion of mucins, the glycoprotein-rich components of the mucus layer that coat the epithelium, providing a physical and biochemical protective barrier against pathogens and mechanical stress (6). Furthermore, *Saccharomyces* and *Lacticaseibacillus* can upregulate the expression of genes associated with barrier integrity, including those encoding tight junction proteins (e.g., occludin, claudins, and zonula occludens), thereby supporting the structural and functional resilience of the intestinal epithelium (6). Collectively, these mechanisms contribute to improved gut homeostasis, enhanced nutrient absorption, and reduced susceptibility to gastrointestinal infections, highlighting the pivotal role of these probiotic species in maintaining gastrointestinal health.

### 2.5 Production of metabolites

*Bifidobacterium* and *Lacticaseibacillus* are prominent probiotic genera that ferment dietary fibers and non-digestible carbohydrates (prebiotics) to produce bioactive metabolites, particularly short-chain fatty acids (SCFAs) such as acetate, propionate, and butyrate (2, 31). These SCFAs play multifaceted roles in maintaining gut health. Butyrate, for example, serves as a primary energy source for colonocytes, supporting intestinal epithelial integrity and barrier function. Acetate and propionate are absorbed systemically and contribute to metabolic regulation, including lipid and glucose homeostasis, while collectively SCFAs help regulate gut motility and pH, creating an environment that suppresses pathogenic bacterial growth (2).

In addition to their metabolic contributions, SCFAs exert significant anti-inflammatory and immunomodulatory effects. They influence the production of regulatory cytokines, enhance mucin secretion, and stimulate the differentiation of regulatory T cells, thereby maintaining intestinal immune homeostasis and protecting against gut-associated inflammation (2, 31). Through these mechanisms, the fermentation activity of *Bifidobacterium* and *Lacticaseibacillus* not only supports nutrient utilization and gut barrier integrity but also contributes to systemic health benefits in the host.

### 2.6 Bile salt hydrolase activity

Members of the genera *Lactobacillus* and *Bifidobacterium* possess the capacity to deconjugate bile salts, an activity that significantly influences bile acid metabolism and the composition of the gut microbiota (2, 31). This deconjugation is mediated by bile salt hydrolase (BSH) enzymes, which hydrolyze conjugated bile acids into free bile acids and amino acids. By modifying the bile acid pool, these probiotics can alter the physicochemical environment of the intestine, affecting lipid digestion, absorption, and microbial colonization patterns (31). The resulting changes in bile acid composition can selectively inhibit pathogenic bacteria while promoting the growth of beneficial microbial populations, thereby contributing to a balanced and resilient gut microbiota (2). Additionally, the modulation of bile acids by BSH-active probiotics has been associated with improved intestinal barrier integrity, enhanced short-chain fatty acid production, and regulation of host lipid and cholesterol metabolism. Collectively, these effects highlight the critical role of *Lactobacillus* and *Bifidobacterium* in maintaining gut health through targeted bile acid modulation (2, 31).

### 2.7 Alteration of gut microbiota composition

**Figure 1:**
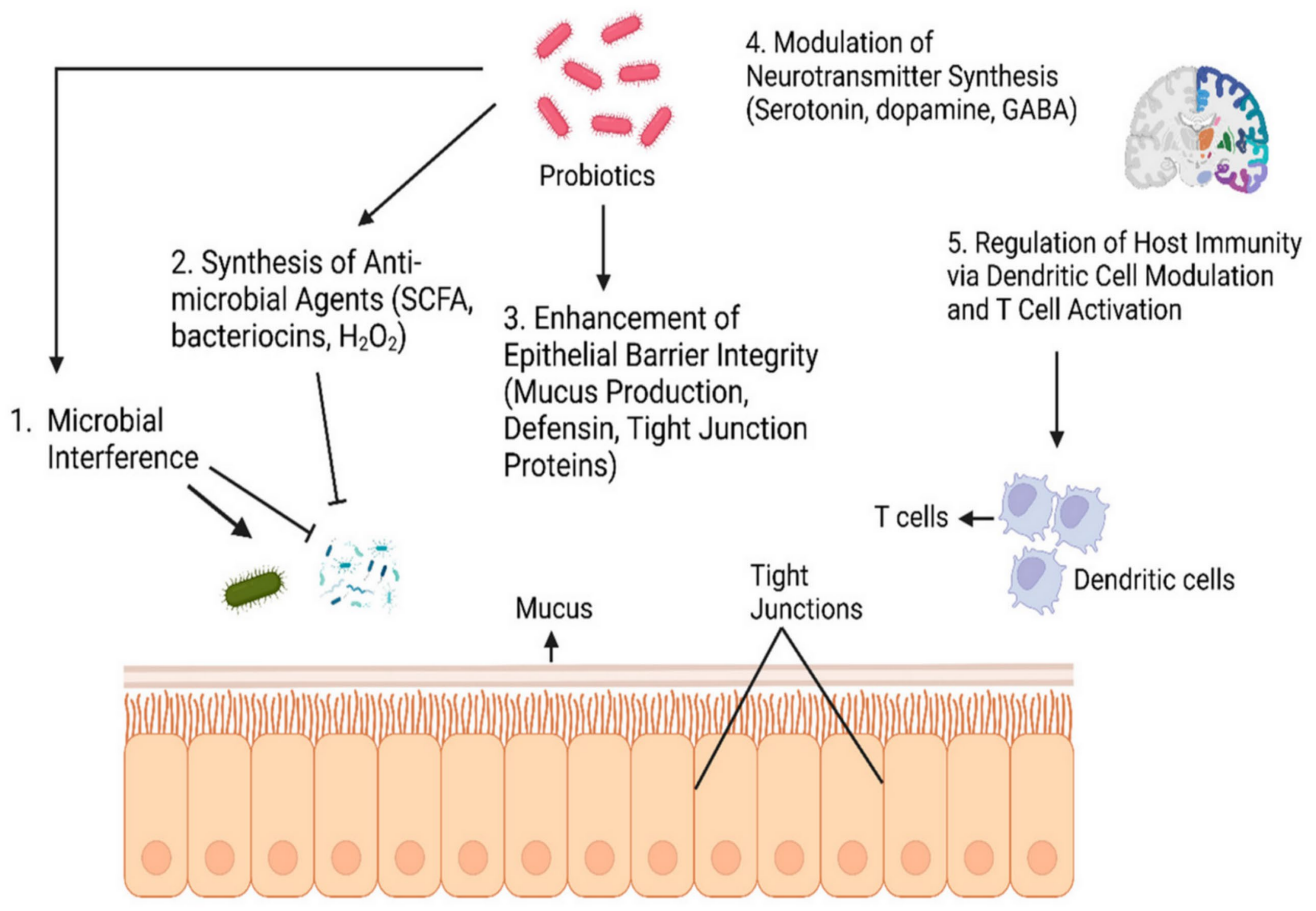
Mechanisms of action of probiotics. Source: Based on work in Naeem and Bourassa (107).

The mechanisms through which probiotics exert these effects are multifaceted and include competitive exclusion of pathogens, production of antimicrobial metabolites (e.g., bacteriocins and short-chain fatty acids), modulation of local pH, enhancement of mucosal barrier integrity, and immunomodulation (2, 33, 35). Additionally, probiotics can influence host metabolic and signalling pathways through interactions with the gut epithelium and the gut–brain axis, thereby extending their benefits beyond the intestine to systemic physiological processes (36). Understanding these diverse mechanisms is critical for the development of targeted probiotic therapies and enables the rational design of formulations aimed at optimizing gut health and mitigating specific disease conditions (Figure 1).

## 3 Effect of probiotics on companion animals’ health

### 3.1 Effect of probiotics in horses

Horses are monogastric herbivores that rely on hindgut fermentation (37), and probiotics are often used to stabilize the complex microbial ecosystem within the large intestine, where billions of microorganisms are responsible for the breakdown of structural carbohydrates (18, 38, 39). Yeast-based probiotics, such as *Saccharomyces cerevisiae*, including strains like NCYC Sc 47 and CNCM I-1077, have been studied in equine nutrition (40). They have been reported to enhance the activity and growth of fiber-digesting bacteria like *Fibrobacter* and *Ruminococcus* species (33, 41). In the hindgut, these yeasts often scavenge oxygen, thereby creating a favorable anaerobic environment for cellulolytic microbes (42). Additionally, bacterial strains such as *Lactobacillus rhamnosus*, *Lactobacillus acidophilus*, *Bifidobacterium animalis, and Lactobacillus plantarum*, are included in some formulations to enhance competitive exclusion of opportunistic pathogens and microbial diversity (43).

Probiotics may also improve feed efficiency and digestion by influencing the degradation of fiber and the stabilization of fermentation. Supplementation with *Saccharomyces cerevisiae* has been linked with increased production of volatile fatty acids, especially propionate and acetate, which serve as major energy sources for horses (40). Certain strains, such as *Lactobacillus reuteri* and *Enterococcus faecium,* assist in regulating lactic acid production and its utilization in the caecum and colon (44). By moderating the fluctuations of pH and minimizing subclinical acidosis in the hindgut, these probiotics may influence the consistent utilization of feed, improve body condition, and potentially enhance athletic performance (45). Some studies also suggest weight gain and feed conversion efficiency improvements in growing horses supplemented with live yeast cultures.

Regarding digestive health, probiotics are frequently administered to alleviate the risk or severity of gastrointestinal disturbances like diarrhea, antibiotic-associated dysbiosis and mild colic. Strains including *Enterococcus faecium*, *Bacillus subtilis and Lactobacillus plantarum*, have been used in equine products to stabilize fecal consistency and mitigate proliferation of pathogenic bacteria (45). Spore-forming species like *Bacillus licheniformis* and *Bacillus subtilis* are vital for enhancing stability in feed and resistance to environmental stress (45). Probiotics may help re-establish commensal populations and reduce the duration of loose manure during or after antibiotic treatment. Yeast supplementation has been incorporated into preventive nutritional strategies to support the stability of fermentation and reduce gas accumulation in horses prone to mild recurrent colic linked to dietary management (41).

Beyond digestion, the gut microbiome plays a vital role in immune regulation, and probiotics may exert immunomodulatory effects via interactions with gut-associated lymphoid tissue (42). *Bifidobacterium longum* and *Lactobacillus rhamnosus* have been associated with improved gut barrier integrity, modulation of inflammatory cytokines, and enhanced mucosal IgA production. The supplementation of probiotics in horses has been explored as a nutrition strategy to improve stability during stressors such as competition, transportation, abrupt environmental changes, or hospitalization (40). By supporting balanced immune signaling and maintaining epithelial barrier function, probiotics may assist in reducing susceptibility to secondary infections and systemic inflammatory responses. While results can vary depending on dosage, strain specificity and individual variability, strategic supplementation of probiotic is recognized as a supportive tool within comprehensive equine health and nutrition programs.

### 3.2 Effect of probiotics in dogs

The use of probiotics in dogs has been reported to relieve diarrhea and general gastrointestinal upset, particularly in cases of antibiotic-associated diarrhea, acute, self-limiting diarrhea, and stress-related digestive disturbances, which could be triggered by dietary change, boarding, or travel (46). *Enterococcus faecium* (strain SF68) has been studied in dogs and shown to decrease the severity and duration of acute diarrhea (47). *Bifidobacterium animalis* (strain AHC7) has also shown some benefits in improving the consistency of faeces and normalizing the frequency of stool (48). Additionally, *Lactobacillus acidophilus*, *Lactobacillus rhamnosus* and *Lactobacillus plantarum* are commonly included in canine probiotic formulations to compete with pathogenic bacteria and enhance microbial balance through mechanisms such as production of antimicrobial substances (bacteriocins), competitive exclusion, and modulation of gut pH (49).

Probiotics are often used as adjunctive therapy in the management of chronic gastrointestinal conditions, in dogs diagnosed with food-responsive enteropathy, inflammatory bowel disease (IBD), or chronic colitis (50). Strains such as *Bifidobacterium animalis*, *Enterococcus faecium,* and *Lactobacillus reuteri* have been incorporated into veterinary probiotic products or therapeutic diets aimed at improving epithelial barrier integrity and modulating intestinal inflammation (51). Some multi-strain formulations containing combinations of *Bifidobacterium*, *Lactobacillus*, and *Streptococcus thermophilus* have been analyzed for their ability to minimize inflammatory markers and increase tight junction protein expression of the intestine. By enhancing mucosal barrier function and balancing immune signaling, probiotics assists to decrease leaky gut, reducing gas production, and alleviating bloating associated with carbohydrate malabsorption or dysbiosis.

The canine immune system is closely linked to the gastrointestinal tract, as a substantial proportion of immune cells reside within the gut-associated lymphoid tissue (GALT). Probiotics influence both innate and adaptive immune responses by interacting with macrophages, dendritic cells, and lymphocytes in the intestinal mucosa. Strains such as *Bifidobacterium longum* and *Lactobacillus rhamnosus* have been linked with companion animal studies to improve phagocytic activity, enhance secretory IgA production and balance cytokine responses (49, 51). The supplementation of specific probiotic strains in the early life of puppies has been investigated for its potential to reduce susceptibility to infections and support immune maturation (46). Additionally, during stressful conditions such as surgery, environmental changes or illness, probiotics may help mitigate stress-induced dysbiosis and secondary immune suppression.

Emerging evidence also supports a role for probiotics in dermatological health and allergy management in dogs (52). The modulation of intestinal microbiota can influence systemic inflammatory responses and skin barrier function because of the gut–skin axis. Certain strains, including *Lactobacillus paracasei*, *Lactobacillus rhamnosus* and *Bifidobacterium animalis*, have been reported for their potential to reduce the severity of atopic dermatitis and pruritus when used alongside conventional therapy (51, 52). These effects are thought to occur through regulation of T-helper cell balance, reduction of pro-inflammatory cytokines, and enhancement of epithelial barrier defenses. Although probiotics are not a primary treatment for allergic skin disease, they can function as an adjunctive therapy within a comprehensive management plan that combines pharmacologic intervention, dietary modification, and environmental control, potentially enhancing clinical outcomes and supporting overall skin health.

### 3.3 Effect of probiotics in cats

In cats, probiotics are increasingly used to support gastrointestinal stability and improve stool quality, particularly in cases of loose stools, mild diarrhea, or recovery following antibiotic therapy (46). Strains such as *Enterococcus faecium* (strain SF68) have been studied in cats and shown to improve the consistency of feaces and reduce the incidence of diarrhea in both household and shelter environments (47). *Bifidobacterium longum* and *Bifidobacterium animalis* are also included in feline probiotic formulations to help restore beneficial microbial populations after dietary disruption or antimicrobial treatment (51). In some cases of mild constipation, probiotics containing *Lactobacillus acidophilus* or *Lactobacillus plantarum* may improve stool regularity via the modulation of fermentation patterns and increasing the production of short-chain fatty acids, which can influence colonic motility (48, 49).

Cats with sensitive stomachs, prone to intermittent vomiting, flatulence, or dietary intolerance, may benefit from probiotic supplementation aimed at enhancing gut barrier integrity and supporting a balanced intestinal microbiota (50). Specific strains, including *Lactobacillus reuteri*, *Lactobacillus rhamnosus*, and *Bifidobacterium bifidum*, have been incorporated into feline supplements to modulate intestinal inflammation and reduce hypersensitivity responses to certain dietary proteins, thereby improving gastrointestinal tolerance and overall digestive health (49, 51). The formulations of multi-strain combining *Enterococcus*, *Lactobacillus*, and *Bifidobacterium* species are sometimes used as adjunctive therapy in cats with food-responsive diarrhea or chronic enteropathy, where modulation of the gut microbiota may alleviate mucosal irritation and enhance the absorption of nutrients (52). The feline immune system is closely linked to the gastrointestinal tract, and probiotics may influence immune responses through interaction with gut-associated lymphoid tissue. Research in kittens, has suggested that supplementation with *Enterococcus faecium* can decrease the severity and duration of upper respiratory tract infections, via the enhancement of secretory IgA production and modulation of mucosal immunity (49). Other strains, including *Bifidobacterium longum*, have been associated with reduced expression of inflammatory cytokines and improved immune signaling balance in companion animals. While probiotics are not a replacement for medical therapy or vaccination, they may provide supportive benefits in maintaining immune stability, especially in young or immunocompromised cats (51, 52).

Probiotics may also play a role in stress management in cats, particularly during events such as rehoming, boarding, introduction into multi-cat households, or other environmental changes. Stress can disrupt microbial balance and alter gut motility, leading to diarrhea, reduced appetite, or behavioral changes (52). Certain strains, such as *Bifidobacterium longum*, have been evaluated for their potential effects on the gut–brain axis, where microbial metabolites influence neurotransmitter pathways and stress responses (51). Probiotics may indirectly support both digestive stability and overall wellbeing by stabilizing the intestinal microbiota during periods of psychological or environmental stress (111). As with other species, probiotic effects in cats are strain-specific and dose-dependent, and clinical responses can vary (49). Selection of products with documented strain identification and evidence of viability is important, and veterinary guidance is recommended when probiotics are used as part of a broader therapeutic plan.

### 3.4 Effects of probiotics in pigs

Probiotics have been extensively investigated as feed additives in pig production to support gastrointestinal health, improve growth performance, and reduce the occurrence of enteric diseases, particularly during the post-weaning period (53, 54). Weaning is a critical stage for piglets because the sudden change from milk to solid feed, separation from the sow, and environmental changes can disrupt the intestinal microbiota and increase susceptibility to digestive disorders. Probiotic preparations commonly used in pigs include bacterial genera such as *Lactobacillus*, *Bacillus*, *Bifidobacterium*, and *Enterococcus*, as well as yeast species like *Saccharomyces cerevisiae* (55–58). These microorganisms can help stabilize the intestinal microbial community by competing with pathogenic bacteria for adhesion sites and nutrients, producing organic acids such as lactic acid, and generating antimicrobial compounds that suppress harmful microorganisms (56).

Several studies have shown that probiotic supplementation may reduce the prevalence and severity of post-weaning diarrhea, which is frequently associated with enterotoxigenic *Escherichia coli* (56, 57). In addition, probiotics have been associated with improvements in intestinal morphology, including increased villus height and a higher villus-to-crypt ratio, both indicators of enhanced nutrient absorption capacity (54, 58). Some experimental and field studies have also reported improvements in feed conversion efficiency, nutrient digestibility, and growth rates when probiotics are included in pig diets (59). Furthermore, probiotics may contribute to immune modulation by stimulating local and systemic immune responses, including increased immunoglobulin production and enhanced immune cell activity (54). However, the effectiveness of probiotics in pigs can vary depending on the microbial strain used, the dosage, the age and health status of the animals and farm management conditions (56).

### 3.5 Effects of probiotics in rabbits

Probiotics have also been explored in rabbit production as a nutritional strategy to maintain intestinal microbial balance and reduce digestive disorders, which are a major cause of morbidity and mortality in intensive rabbit farming systems (15). Rabbits possess a specialized digestive system characterized by hindgut fermentation, where a large and complex microbial population in the cecum plays an essential role in fiber digestion and nutrient utilization (16). Because of this delicate microbial ecosystem, rabbits are particularly sensitive to disturbances in gut microbiota, which can lead to enteric diseases such as enteritis and diarrhea (60). Probiotic microorganisms evaluated in rabbit diets commonly include species of *Lactobacillus*, *Bacillus*, *Enterococcus*, and yeast such as *Saccharomyces cerevisiae* (61, 62).

These microorganisms may help stabilize the intestinal microbial environment by promoting the growth of beneficial bacteria and limiting the proliferation of pathogenic microorganisms (60). Research studies have reported that probiotic supplementation can improve feed utilization and growth performance in rabbits by enhancing digestive efficiency and supporting the fermentation processes occurring in the cecum (63). Some investigations have also shown that probiotics may positively influence intestinal morphology and gut barrier function, which can contribute to improved nutrient absorption and overall digestive health (61, 64). In addition, probiotics have been studied for their potential role in modulating immune responses in rabbits, including stimulation of immune cells and increased resistance to certain infections (64). There is also evidence from some experimental studies suggesting that probiotic supplementation may influence oxidative status by reducing certain markers of oxidative stress (16, 65). However, as with pigs, the observed benefits depend on factors such as the probiotic strain, dosage, diet composition, and environmental conditions. Generally, probiotics are being increasingly investigated as a potential non-antibiotic feed additive for supporting health and productivity in rabbit production systems (16).

## 4 Overview of oxidative stress and its impact on monogastric animals

Oxidative stress arises when the production of reactive oxygen species (ROS) exceeds the capacity of the organism to neutralize these reactive intermediates or repair the resulting cellular damage (26, 74). ROS are highly reactive molecules, including free radicals such as superoxide anion (O₂^−^), hydroxyl radicals (OH•), and non-radical species like hydrogen peroxide (H₂O₂), which can damage key cellular components, including lipids, proteins, and DNA (6). Mitochondrial respiration is a principal endogenous source of ROS, where incomplete reduction of oxygen during the electron transport chain generates superoxide radicals (28). In addition, activated immune cells produce ROS as part of the host defense during inflammatory responses. Environmental and dietary factors—such as high-fat diets, excessive intake of pro-oxidant nutrients, or deficiency of dietary antioxidants—can further exacerbate ROS production and oxidative stress (28).

Monogastric animals and humans, are particularly susceptible to oxidative stress due to their high metabolic rates and limited capacity to detoxify ROS compared with ruminants (6, 26, 74). Oxidative stress can compromise growth by damaging enterocytes and other cells critical for nutrient absorption and metabolism, reducing protein synthesis efficiency, and impairing muscle development (34). Damage to the intestinal epithelium can diminish nutrient uptake, negatively affecting feed conversion and overall performance. Moreover, oxidative stress can adversely affect reproductive tissues, leading to sperm DNA damage in males, reduced oocyte quality in females, and, in pregnant animals, complications such as preterm birth or developmental impairments in offspring (28, 74). Chronic oxidative stress can also impair immune function, rendering animals more vulnerable to infections and disease. It diminishes the production and activity of protective immune cells and antibodies, while excessive ROS can trigger sustained inflammatory responses, contributing to conditions such as arthritis, enteritis, and other inflammatory disorders (28, 74). At the cellular level, ROS initiate lipid peroxidation, compromising membrane integrity and function, which can lead to cell death and tissue damage (6). Proteins are susceptible to oxidation, resulting in loss of enzymatic activity, structural alterations, and accumulation of dysfunctional proteins. DNA damage caused by ROS, including strand breaks and mutations, can increase the risk of cancer or heritable genetic defects.

**Figure 2:**
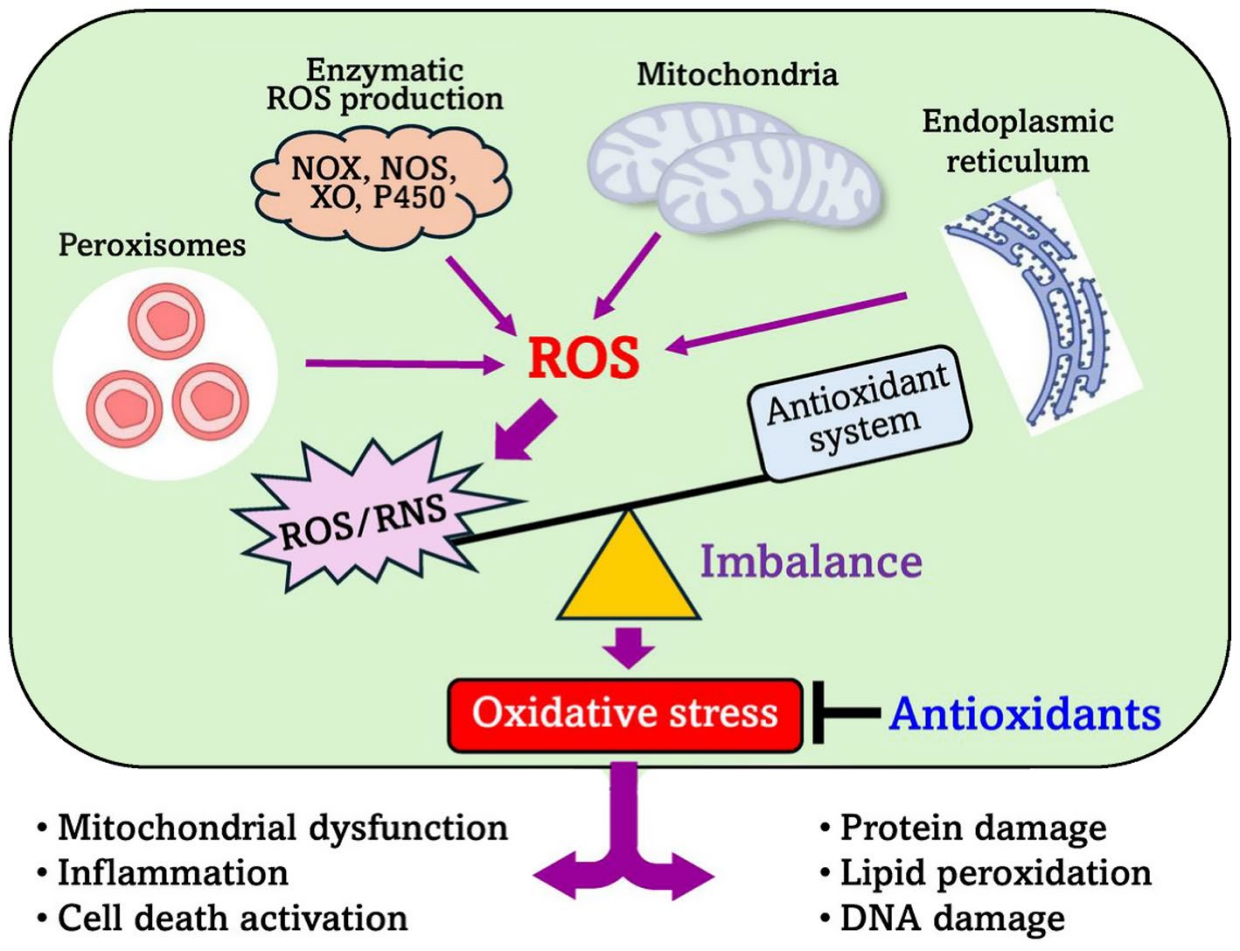
Antioxidant defence system. Enzymatic ROS production induces oxidative stress. Dietary interventions, including antioxidants (like probiotics), can prevent this system imbalance. Source: Based on work in Surai et al. (108).

Monogastric animals possess endogenous antioxidant defense systems to counteract ROS, including enzymatic antioxidants such as superoxide dismutase (SOD), catalase, and glutathione peroxidase. However, these defenses can be overwhelmed under conditions of excessive ROS generation, leading to a state of oxidative stress (34) (Figure 2). Understanding the sources, impacts, and defense mechanisms of oxidative stress is therefore critical for developing strategies—such as dietary antioxidant supplementation or probiotic interventions—to protect animal health and optimize productivity.

### 4.1 Types of antioxidants produced by probiotics

Probiotics contribute to host antioxidant defense through multiple, interconnected mechanisms, thereby reducing oxidative stress, supporting gut health, and potentially conferring systemic health benefits (66, 67). Their ability to produce or enhance antioxidant molecules underscores a role that extends beyond intestinal wellbeing, influencing overall oxidative balance and contributing to disease prevention (68).

#### 4.1.1 Phenolic compounds

Certain probiotic genera, including *Saccharomyces* and *Bifidobacterium* can produce or enhance the generation of phenolic compounds, which are potent antioxidants (5). Phenolic compounds neutralize free radicals, mitigate oxidative stress, and protect cellular macromolecules from damage. For example, *Lacticaseibacillus rhamnosus* has been reported to enhance phenolic content in fermented foods, contributing to their antioxidant potential (31).

#### 4.1.2 Vitamin production

Probiotic strains such as *Lacticaseibacillus rhamnosus,* and *Bifidobacterium bifidum* can synthesize vitamins with antioxidant activity, including vitamin B12 and folate (3). These vitamins participate in critical biochemical processes such as DNA synthesis and methylation, thereby reducing oxidative damage. While probiotics can produce B vitamins in the gut, the amounts and bioavailability are influenced by competition with other microbes and gut integrity (2, 31).

#### 4.1.3 Glutathione synthesis

Glutathione, a key intracellular antioxidant, neutralizes free radicals and reactive oxygen species (ROS), regenerates other antioxidants, and detoxifies harmful compounds (34) Strains of *Saccharomyces, Lacticaseibacillus,* and *Bifidobacterium* have been shown to contribute to glutathione synthesis or regeneration within the gut, enhancing cellular defense against oxidative damage (2, 31, 34).

#### 4.1.4 Antioxidant enzymes

Probiotics also produce enzymatic antioxidants. Superoxide dismutase (SOD) catalyzes the conversion of superoxide radicals into hydrogen peroxide and oxygen, thereby reducing oxidative stress and preventing cellular injury (69). *Saccharomyces* and *Lacticaseibacillus* genera are known producers of SOD (28, 31, 70). Catalase further detoxifies hydrogen peroxide into water and oxygen, mitigating potential oxidative damage; species such as *Saccharomyces cerevisiae* and *Lacticaseibacillus reuteri* are notable producers (31, 34). Additionally, peroxidases reduce various peroxides to less reactive compounds, further contributing to the host’s antioxidant capacity (6, 69).

#### 4.1.5 Bacteriocins and indirect antioxidant effects

Probiotic-derived bacteriocins, primarily recognized for their antimicrobial activity, can also exert indirect antioxidant effects by modulating the gut microbiota and preventing the accumulation of pro-oxidant by-products (21, 71). *Lacticaseibacillus* species produce diverse bacteriocins that not only suppress pathogenic microbes but also reduce inflammation and oxidative stress in the gastrointestinal tract (21, 71).

Collectively, these mechanisms illustrate the multifaceted antioxidant roles of probiotics, encompassing direct free radical scavenging, enzymatic detoxification, micronutrient synthesis, and modulation of the gut microbial ecosystem. These properties highlight their potential in maintaining oxidative homeostasis and supporting systemic health in monogastric animals.

## 5 Probiotics as antistress and growth promoting agent

### 5.1 Impact of probiotics on the gut-brain axis and stress response

The gut-brain axis is a bidirectional communication system linking the gut and the brain, influencing various aspects of mental health and stress response (3). Probiotics impact this axis in several ways, thereby affecting stress levels and emotional wellbeing.

#### 5.1.1 Neurotransmitter production

Probiotics, particularly *Lacticaseibacillus rhamnosus*, can influence the production and regulation of key neurotransmitters in the gut, including serotonin, dopamine, and gamma-aminobutyric acid (GABA) (72). These neurotransmitters are central to mood regulation, stress response, and overall nervous system function. Notably, approximately 90–95% of the body’s serotonin is synthesized in the gastrointestinal tract (71). Probiotics can enhance the availability of tryptophan, the biochemical precursor to serotonin, thereby potentially elevating systemic serotonin levels (66). Elevated serotonin contributes to anxiolytic and calming effects on the central nervous system, helping modulate behavioral and physiological responses to stress (31). Additionally, microbial-derived GABA and dopamine can further influence neurochemical signaling, contributing to improved stress resilience and emotional stability (72).

#### 5.1.2 Hypothalamus–pituitary–adrenal (HPA) axis regulation

The hypothalamus–pituitary–adrenal (HPA) axis is the central neuroendocrine system responsible for coordinating stress responses through the release of corticotropin-releasing hormone (CRH), adrenocorticotropic hormone (ACTH), and cortisol (corticosterone in animals) (69). Certain probiotics, including *Saccharomyces cerevisiae*, can modulate HPA axis activity, influencing cortisol production and attenuating hyperactivation of the stress response (71). By regulating HPA signaling pathways, probiotics reduce excessive glucocorticoid release, which in turn mitigates the physiological and behavioral impacts of chronic stress (67). This regulatory effect supports enhanced resilience to environmental and physiological stressors, with potential benefits for both mental and physical health.

#### 5.1.3 Microbial diversity

Probiotic strains such as *Bacillus subtilis, Lacticaseibacillus rhamnosus,* and *Saccharomyces cerevisiae* contribute to the maintenance of a balanced gut microbiota by promoting beneficial bacterial populations and inhibiting pathogenic microorganisms (27). A diverse and stable microbiome is critical for effective gut–brain communication. Probiotics influence the production of microbial metabolites, including short-chain fatty acids (SCFAs), which can cross the gut–brain interface and modulate neural and hormonal pathways involved in stress and cognition (23). Moreover, probiotics enhance gut barrier integrity, preventing translocation of harmful metabolites and endotoxins that could trigger systemic inflammation and adversely affect brain function (73).

#### 5.1.4 Anti-inflammatory effects

Chronic stress is often accompanied by systemic inflammation, which can impair brain function, alter neurotransmitter signaling, and negatively influence mood (74). Probiotics such as *Saccharomyces cerevisiae* can modulate immune responses, reducing the production of pro-inflammatory cytokines like tumor necrosis factor-alpha (TNF-α) and interleukin-6 (IL-6), while enhancing anti-inflammatory cytokine expression (8). By mitigating inflammation and supporting intestinal health, probiotics contribute to improved stress resilience and emotional wellbeing (6, 8, 71). This anti-inflammatory action may also indirectly support HPA axis regulation and neurotransmitter homeostasis.

#### 5.1.5 Vagus nerve stimulation

The vagus nerve serves as a principal communication conduit between the gut and the central nervous system (114). *Lacticaseibacillus rhamnosus* and similar probiotic strains can influence vagal signaling by modulating the production and release of neurotransmitters and neuroactive metabolites in the gut. These signals travel via the vagus nerve to the brain, affecting stress responses, mood regulation, and the entire emotional state (114). Vagus-mediated pathways thus represent a critical mechanism by which probiotics exert psychobiotic effects and support gut–brain homeostasis.

### 5.2 Influence on digestion and nutrient absorption

**Figure 3:**
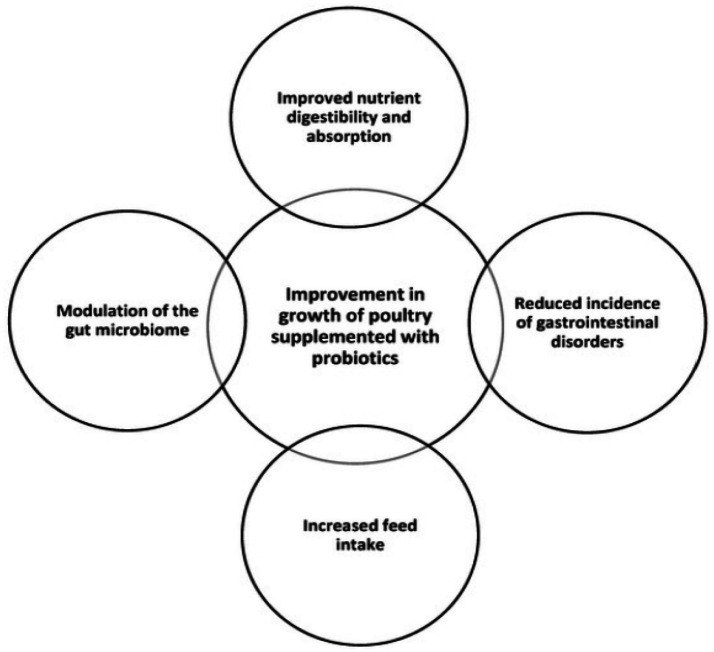
Influence of probiotics on digestion and nutrient absorption. Source: Adapted from Naeem and Bourassa (107), published under CC BY 4.0 license.

Probiotics such as *Saccharomyces cerevisiae, Bifidobacterium bifidum,* and *Lacticaseibacillus rhamnosus* are increasingly employed as complementary strategies in clinical nutrition to support digestion and nutrient absorption, particularly in individuals or animals with gastrointestinal disturbances or following antibiotic therapy (34, 72). These microorganisms improve gastrointestinal function, alleviate digestive discomfort, and promote overall digestive health by enhancing gastrointestinal motility and regulating intestinal peristalsis (25, 27, 75). Mechanistically, probiotics modulate the secretion of gut hormones and neurotransmitters, facilitating efficient transit of food and waste through the digestive tract. Probiotics contribute to nutrient digestion by enhancing the activity of digestive enzymes. They produce or stimulate the production of proteases, lipases, and amylases, thereby improving the breakdown and absorption of proteins, fats, and carbohydrates (76). Additionally, probiotics protect the gut from pathogenic microorganisms by competing for adhesion sites and resources, as well as producing antimicrobial compounds that inhibit pathogen proliferation (29, 77). These actions collectively reduce gastrointestinal infections, enhance gut barrier integrity, and optimize nutrient absorption (20). Probiotic-mediated production of short-chain fatty acids (SCFAs) and antimicrobial peptides reinforces tight junctions between intestinal epithelial cells, improving gut permeability and preventing the translocation of undigested food particles and toxins into the bloodstream (20, 75, 78, 79) (Figure 3).

#### 5.2.1 Maintenance of gut health and microbiota balance

Beyond digestion, probiotics play a pivotal role in maintaining gut microbial balance and overall gut health (80). They help restore and preserve microbial diversity, particularly after disruptions caused by antibiotic use, dietary changes, or gastrointestinal diseases (9). By introducing beneficial microbes, probiotics repopulate the gut with commensals that compete with pathogens for nutrients and adhesion sites while producing antimicrobial substances that inhibit pathogen colonization (29, 112). Different probiotic strains confer distinct benefits in monogastric animals. Commonly used strains include *Saccharomyces cerevisiae, Bifidobacterium bifidum, Lacticaseibacillus rhamnosus, Enterococcus faecium,* and *Bacillus subtilis*, which improve nutrient absorption, gut health, and growth performance (28, 34, 80, 81).

For example, *Saccharomyces* species produce antimicrobial compounds, enhance gut barrier function, reduce gastrointestinal inflammation, and alleviate heat stress effects (6, 34, 35, 82). *Lacticaseibacillus rhamnosus* maintains a balanced microbiota, lowers gut pH through lactic acid production, inhibits pathogens, improves feed conversion, enhances immune responses, and competes with pathogenic bacteria for adhesion sites (70, 83).

*Bifidobacterium bifidum* produces SCFAs, improves digestion and lactose tolerance, enhances immune function, and supports balanced gut microbiota (84). *Enterococcus faecium* secretes antimicrobial compounds, competes with pathogens, and promotes growth performance while reducing gastrointestinal diseases such as diarrhoea (85). *Bacillus subtilis* produces enzymes aiding feed digestion, competes with pathogens, enhances feed conversion, improves nutrient absorption, and produces beneficial metabolites supporting gut health (84–86). Combination probiotics, containing multiple strains, are designed to deliver synergistic benefits, simultaneously enhancing gut health, immunity, and growth performance (84, 86). Table 1 summarizes key probiotic strains and their documented effects in monogastric animals.

**Table 1:** Commonly used probiotics in monogastric production.

Probiotics	Description	References
*Saccharomyces cerevisiae* (yeast)	They assist in the process of fermenting indigestible materials in the gut. *S. cerevisiae* produces bioactive compounds during fermentation, such as prebiotic oligosaccharides and *β*-glucans, which enhance the immune response and promote gut health.	(33, 34)
*Bifidobacterium* species	*Bifidobacterium longum, Bifidobacterium animalis and Bifidobacterium bifidum,* etc. are responsible for modulating immune responses, they also decrease the load of pathogens.	(87)
*Bacillus* species	*Bacillus subtilis, Bacillus coagulans and Bacillus cereus*. *Bacillus ensures* stability and resilience in feed due to its spore-forming capability.	(9, 85)
*Enterococcus* species	*Enterococcus faecium*. They are responsible for the production of antimicrobial peptide that enhances and inhibits pathogen proliferation.	(81)
*Streptococcus* species	They contribute to gut health by modulating intestinal microbiota and producing SCFAs.	(115)
*Lactobacillus* species	They improve the general performance and improve gut health.	(83)

## 6 General safety considerations

The safety evaluation of probiotics in monogastric animals is critical to prevent adverse effects and ensure reliable benefits (87). Safety assessment involves examining potential toxicity, side effects, and overall suitability of probiotic strains for species such as poultry and swine (88).

### 6.1 Toxicity and adverse effects

Probiotics are generally regarded as safe and non-toxic. However, excessive dosages may disrupt the natural gut microbiota or induce mild gastrointestinal disturbances such as bloating or diarrhoea, particularly when introducing strains like *Saccharomyces cerevisiae OYR-481* or *Lacticaseibacillus rhamnosus GG* (89, 113). There is probably a very high risk of contaminating bacteria, which could also be pathogenic (89). These effects are typically transient, resolving as the gut microbiota adapts to the new microbial populations (89, 90).

### 6.2 Regulatory and safety assessments

GRAS Status: Certain strains, including *Lacticaseibacillus rhamnosus GG* and *Saccharomyces cerevisiae OYR-481*, are recognized as Generally Recognized As Safe (GRAS) by the U.S. FDA, indicating historical or scientifically verified safety (89). European Food Safety Authority (EFSA): EFSA evaluates probiotic strains for use in animal feed, assessing safety, efficacy, and potential risks before commercial approval (90, 91). Pre-clinical Trials: Probiotics undergo pre-clinical trials to assess safety, efficacy, and potential adverse effects in controlled conditions before widespread use (77, 92, 115).

#### 6.2.1 Strain-specific safety

*Saccharomyces cerevisiae OYR-481* is well-characterized and safe when used at recommended doses (90). *Lacticaseibacillus rhamnosus GG* is GRAS, though rare infections can occur in immunocompromised animals (90). *Bifidobacterium longum 1714* is GRAS with minimal reported adverse effects (90). *Enterococcus faecium UC7251* is generally safe; some species are opportunistic pathogens, highlighting the importance of strain selection and hygiene (90). *Bacillus subtilis str. AUSI98* is generally safe, but monitoring for minor gastrointestinal disturbances is advisable (80).

### 6.3 Monitoring and quality control

Maintaining probiotic safety requires adherence to recommended dosages, correct strain identification, and rigorous quality control to prevent contamination (89, 91). Verification through molecular methods and monitoring of animal health, growth performance, and gastrointestinal status ensures continued efficacy and safety (91, 93).

### 6.4 Practical recommendations

Probiotics should be introduced gradually, starting at lower doses to allow gut adaptation. Animals should be monitored for adverse reactions during initial administration (90). Products from reputable manufacturers with detailed strain information and quality assurance are recommended (94).

### 6.5 Side effects

While generally safe, probiotics may occasionally cause transient gastrointestinal discomfort or interact with medications, emphasizing the need for veterinary oversight and adherence to recommended dosages (95–100).

### 6.6 Regulatory bodies and their roles

The FDA and CVM: Regulate probiotics in animal feed, ensuring GRAS status, safety, efficacy, and accurate labeling (88, 89). European Food Safety Authority (EFSA): Assesses probiotic safety and efficacy in the EU, authorizing only strains with thorough evaluation (90). Joint Expert Committee on Food Additives (JECFA): Provides international safety guidelines for probiotics in animal feed and human consumption, helping harmonize regulations (22, 90, 97).

## 7 Dosage recommendations

Determining appropriate dosage and administration methods for probiotics in monogastric animals is critical to ensure their efficacy while minimizing potential risks (28). Effective probiotic use depends on several factors, including the genus, species, specific strain, the species of animal, the age and physiological status of the animals, and the intended health or production outcomes (101). Probiotic doses are typically expressed in colony-forming units (CFUs), and effective dosages can range widely depending on the strain and desired effect, commonly between 1 × 10^6^ and 1 × 10^10^ CFUs per animal per day (3). Different animal species and developmental stages require tailored dosages, and guidance from manufacturers or veterinary professionals should always be followed (90).

For poultry, recommended doses usually range from 1 × 10^8^ to 1 × 10^9^ CFUs per bird per day, with adjustments based on age, weight, and production goals (102). In pigs, growing animals typically receive 1 × 10^9^ to 1 × 10^10^ CFUs per animal per day, while piglets may require lower doses of 1 × 10^8^ to 1 × 10^9^ CFUs per animal per day (93, 103, 104). Gradual introduction starting with lower doses and incrementally increasing to the recommended level helps the gut microbiota adapt and reduces the risk of adverse effects in animals.

### 7.1 Mode of administration of probiotics

Probiotics can be administered through several practical routes:

Feed supplementation: Probiotics can be thoroughly mixed into complete feed, ensuring uniform distribution so that each animal receives an accurate dose (28). Drinking water: Particularly in poultry and aquaculture, probiotics can be administered through drinking water, providing an easy method of intake and ensuring rapid delivery to the gastrointestinal tract (81). Direct oral administration: Small animals or individual treatments can utilize dosing syringes or other tools for precise delivery (94). Top-dressing: Probiotics may be sprinkled over feed, offering a flexible approach to dosing and ensuring consumption alongside feed intake. The choice of administration method depends on factors such as animal size, management system, ease of application, and stability of the probiotic under different conditions.

### 7.2 Assessment of current feed programs

Integrating probiotics into existing feed programs requires careful evaluation to maximize benefits and complement overall nutrition strategies (94). Existing feed formulations should be reviewed to confirm that they meet the nutritional requirements of the animals and to identify any gaps that probiotics could help address (112). Probiotics should be incorporated strategically to achieve specific objectives such as improving gut health, enhancing nutrient absorption, supporting growth performance, or mitigating stress-related impacts (22, 72). Proper integration also involves monitoring feed intake, growth performance, and health indicators after probiotic supplementation to assess effectiveness and make adjustments as needed. A well-planned probiotic program works synergistically with feed and management practices to optimize productivity and animal wellbeing.

## 8 Future directions and research needs

Despite substantial progress in probiotic research, several knowledge gaps remain, highlighting critical areas for further investigation to enhance the efficacy, safety, and application of probiotics. A major research need is comprehensive profiling of individual probiotic strains across diverse health contexts. While many studies focus on a limited number of strains, outcomes often vary, emphasizing the necessity of detailed mechanistic studies to elucidate how specific strains interact with host cells, gut microbiota, and immune pathways at the molecular level (6). Furthermore, long-term effects of probiotic supplementation remain underexplored. Longitudinal studies are essential to evaluate sustained impacts on health, microbiota stability, chronic disease progression, and potential adverse effects (27, 102).

The complex interactions between probiotics and the existing gut microbiota are not fully understood. Future research should investigate how probiotics alter microbial diversity and function, and how these changes translate into measurable health outcomes (29). Individual variability arising from genetic, dietary, and environmental factors also influences probiotic efficacy and warrants further study.

Improving the stability and delivery of probiotics is another priority. Research should focus on developing advanced encapsulation methods, optimizing storage conditions, and designing delivery systems that ensure probiotics reach their target sites in the gastrointestinal tract (28). Evaluation of synbiotics (combinations of probiotics and prebiotics) and other innovative formulations across different health contexts is also needed.

There is a pressing need for large-scale, high-quality clinical trials to validate probiotic efficacy for specific conditions, including gastrointestinal disorders, metabolic syndrome, and mental health challenges (3, 5, 12, 27, 34, 76, 109). Standardized labeling practices, manufacturing quality control, and regulatory guidelines are crucial to ensure product purity, potency, and consumer confidence. Additionally, exploring interactions between probiotics and other therapeutic agents, such as antibiotics and immunotherapies, can reveal synergistic or antagonistic effects (10, 14).

Finally, research should examine integration of probiotics into broader health strategies, considering diet, lifestyle, and medical interventions, as well as their cost-effectiveness and accessibility (23, 31, 110). Understanding public perceptions and improving education around probiotics will support informed decision-making and enhance the practical impact of probiotic interventions.

### 8.1 Potential for new probiotic formulations and applications

Advances in microbiology, biotechnology, and gut microbiome research offer vast potential for the development of novel probiotic formulations and applications. Discovery and characterization of less-studied or novel strains with unique therapeutic properties could enable targeted interventions for immune modulation, stress reduction, or other health outcomes (105).

Combination probiotics, comprising multiple complementary strains, can simultaneously address diverse aspects of gut health and overall wellbeing. Microencapsulation and nano-encapsulation technologies can enhance probiotic survival through the gastrointestinal tract, enabling controlled and targeted release to maximize efficacy and minimize side effects (33). Novel delivery formats—such as capsules, powders, or chewables—can improve palatability, convenience, and compliance across different populations. Personalized probiotic approaches, leveraging individual microbiome profiles and genetic information, could allow for tailored interventions that align with unique gut microbial compositions and genetic predispositions (106). This precision approach promises to optimize therapeutic outcomes while minimizing risks.

Finally, providing transparent information on probiotic strains, formulations, and expected health benefits is essential to empower consumers, farmers, and clinicians to make informed choices, thereby enhancing the adoption and impact of probiotic-based strategies in both human and animal health.

## 9 Conclusion

Probiotics enhance health, gut stability, and productivity in monogastric and companion animals through antioxidant, antistress, and growth-promoting effects. They reduce oxidative stress, improve nutrient digestion, modulate the gut–brain axis, and support immune function. Their effectiveness depends on strain, dosage, delivery method, and host species.

Recommended supplementation for monogastric animals: 10^8^–10^9^ CFU/g feed for bacterial probiotics in pigs, 10^7^–10^9^ CFU/g feed in rabbits, and 2–5 g/kg feed for yeast-based probiotics is considered safe for horses and poultry. Excessive use provides no additional benefit and may disrupt gut microbiota. Careful, species-specific dosing and integration into nutrition and management programs offer a sustainable approach to improving animal health, productivity, and welfare. Continued research is needed to optimize mechanisms, dosage, and synergistic effects with other dietary components.

## References

[1] Atuahene D Sam BA Idan F Sana SS Knop R Suthar T . Probiotics, prebiotics, and synbiotics in pigs and poultry: a review of gut health, performance, and environmental outcomes. Vet Sci. (2025) 12:1054. doi: 10.3390/vetsci12111054, 41295692 PMC12656940

[2] Limbu D Sarkar BR Adhikari MD. "Role of probiotics and prebiotics in animal nutrition". In: Sustainable Agriculture Reviews: Animal Biotechnology and Livestock Production, vol. 4. Cham: Springer (2024). p. 173–204.

[3] Shaffi MS Hameed MK. The role of probiotics in animal nutrition and health. World J Adv Res Rev. (2023) 17:276–80.

[4] Tang X Zeng Y Xiong K Zhong J. *Bacillus* spp. as potential probiotics promoting piglet growth by improving intestinal health. Front Vet Sci. (2024) 11:1429233. doi: 10.3389/fvets.2024.1429233, 39132437 PMC11310147

[5] Mia N Alam AMMN Rahman MM Ali MS Hashem MA. Probiotics to enhance animal production performance and meat quality: a review. Meat Res. (2024) 4:21–9. doi: 10.55002/mr.4.2.85

[6] Sumanu VO Byaruhanga C Bosman AM Ochai SO Naidoo V Oosthuizen MC . Effects of *Saccharomyces cerevisiae* probiotic and ascorbic acid on oxidative gene damage biomarker, heat shock protein 70 and interleukin-10 in broiler chickens exposed to heat stress. Anim Gene. (2023) 28:200150

[7] Ruampatana J Suwimonteerabutr J Homyog K Mekboonsonglarp W Kanjanavaikoon K der Veken WV . *Clostridium butyricum* probiotic feed additive: modulation of sow milk metabolomics and mitigation of pre-weaning piglet diarrhea. Animals. (2024) 14:2098. doi: 10.3390/ani14142098, 39061560 PMC11273528

[8] Sampath V Cho S Jeong J Mun S Lee CH Hermes RG . Dietary *Bacillus* spp. supplementation to both sow and progenies improves post-weaning growth rate, gut function, and reduces pro-inflammatory cytokine production in weaners challenged with *Escherichia coli* K88. Anim Microbiome. (2024) 6:3–12. doi: 10.1186/s42523-024-00290-y, 38268054 PMC10809626

[9] Bahaddad SA Almalki MH Alghamdi OA Sohrab SS Yasir M Azhar EI . *Bacillus* species as direct-fed microbial antibiotic alternatives for monogastric production. Probiotics Antimicrob Proteins. (2023) 15:1–16. doi: 10.1007/s12602-022-09909-5, 35092567 PMC8799964

[10] Rabetafika HN Razafindralambo A Ebenso B Razafindralambo HL. Probiotics as antibiotic alternatives for human and animal applications. Encyclopedia. (2023) 3:561–81. doi: 10.3390/encyclopedia3020040

[11] Ding S Yan W Ma Y Fang J. The impact of probiotics on gut health via alteration of immune status of monogastric animals. Anim Nutr. (2021) 7:24–30. doi: 10.1016/j.aninu.2020.11.004 33997328 PMC8110871

[12] Slizewska K Chlebicz-Wojcik A Nowak A. Probiotic properties of new *Lactobacillus* strains intended for use as feed additives for monogastric animals. Probiotics Antimicrob Proteins. (2021) 13:146–62. doi: 10.1007/s12602-020-09674-3, 32577907 PMC7904557

[13] Bhogoju S Nahashon S. Recent advances in probiotic application in animal health and nutrition: a review. Agriculture. (2022) 12:304. doi: 10.3390/agriculture12020304

[14] Saettone V Biasato I Radice E Schiavone A Bergero D Meineri G. State-of-the-art of nutritional alternatives to the use of antibiotics in humans and monogastric animals. Animals. (2020) 10:2199. doi: 10.3390/ani10122199 33255356 PMC7759783

[15] Anas H Mohamed MA Hassan RI Gomaa WM Mustafa FEZA. *Lactobacillus plantarum* and *Lactobacillus acidophilus* enhance growth performance, immunity, cecal microbiota, and vital organs histomorphology in rabbits. Sci Rep. (2026) 16:3268. doi: 10.1038/s41598-025-33763-4, 41577854 PMC12835538

[16] Ebeid TA Saleh AA El-Ratel IT Alkhalaf AN Mousa EF. The beneficial effects of probiotics on rabbit’s productivity and health–a review. Ann Anim Sci. (2026) 26:189–99. doi: 10.2478/aoas-2025-0043

[17] Sampath V Sureshkumar S Kim IH. The efficacy of yeast supplementation on monogastric animal performance—a short review. Life. (2023) 13:2037. doi: 10.3390/life13102037, 37895419 PMC10608604

[18] Schoster A Weese JS Guardabassi L. Probiotic use in horses–what is the evidence for their clinical efficacy? J Vet Intern Med. (2014) 28:1640–52. doi: 10.1111/jvim.12451, 25231539 PMC4895607

[19] Sugiharto S Yudiarti T Isroli I Widiastuti E Kusumanti E. Dietary supplementation of probiotics in poultry exposed to heat stress: a review. Ann Anim Sci. (2017) 17:591–604. doi: 10.1515/aoas-2016-0062

[20] Luise D Bosi P Raff L Amatucci L Virdis S Trevisi P. *Bacillus* spp. probiotic strains as a potential tool for limiting the use of antibiotics and improving the growth and health of pigs and chickens. Front Microbiol. (2022) 13:801827. doi: 10.3389/fmicb.2022.801827, 35197953 PMC8859173

[21] Melara EG Avellaneda MC Valdivie M Garcia-Hernandez Y Aroche R Martinez Y. Probiotics: symbiotic relationship with the animal host. Animals. (2022) 12:719. doi: 10.3390/ani12060719, 35327116 PMC8944810

[22] Zoumpopoulou G Kazou M Alexandraki V Angelopoulou A Papadimitriou K Pot B . "Probiotics and prebiotics: an overview on recent trends". In: Probiotics and Prebiotics in Animal Health and Food Safety. Cham: Springer (2018). p. 1–34.

[23] Al-Otaibi AM Abd El-Hack ME Dmour SM Alsowayeh N Khafaga AF Ashour EA . Narrative review on the beneficial impacts of probiotics on poultry: an updated knowledge. Ann Anim Sci. (2023) 23:405–18.

[24] Barba-Vidal E Martin-Orue SM Castillejos L. Practical aspects of the use of probiotics in pig production: a review. Livest Sci. (2019) 223:84–96. doi: 10.1016/j.livsci.2019.02.017

[25] Dermyshi E Wang Y Yan C Hong W Qiu G Gong X . The “golden age” of probiotics: a systematic review and meta-analysis of randomized and observational studies in preterm infants. Neonatology. (2017) 112:9–23. doi: 10.1159/000454668, 28196365

[26] Aluwong T Sumanu VO Ayo JO Ocheja B Zakari F Minka N. Daily rhythms of cloacal temperature in broiler chickens of different age groups administered zinc gluconate and probiotic during the hot-dry season. Physiol Rep. (2017) 5:e13314. doi: 10.14814/phy2.13314 28637707 PMC5492204

[27] Soto-Diaz A Rondon-Castillo AJ Iglesias-Gomez JM. Probiotics in animal production: action mechanisms and beneficial effects on animal husbandry. Pastos Forrajes. (2023) 46:25–34.

[28] Sumanu VO Naidoo V Oosthuizen MC Chamunorwa JP. Adverse effects of heat stress during summer on broiler chickens production and antioxidant mitigating effects. Int J Biometeorol. (2022) 66:2379–93. doi: 10.1007/s00484-022-02372-5 36169706

[29] Perez-Guerra N Fafando-Bernardez P Mendez J Cachaldora P Pastrana-Castro L. Production of four potentially probiotic lactic acid bacteria and their evaluation as feed additives for weaned piglets. Anim Feed Sci Technol. (2007) 134:89–107.

[30] Vieco-Saiz N Lemâle O Evans N Quinteiro-Filho WM Mellouk A Consuegra J . Winning the battle of intestinal peace with *Bacillus*: a multifaceted approach to animal health, immunity and future applications in monogastric livestock production. Front Microbiol. (2025) 16:1711747. doi: 10.3389/fmicb.2025.1711747, 41472814 PMC12747676

[31] Omar MA. Economic evaluation of probiotic (*Lactobacillus acidophilus*) use in different broiler breeds in Egypt. Benha Vet Med J. (2014) 26:52–60.

[32] Buonaiuto G Danese T El-Sabrout K Yıldırım A. Bioactive feed additives in animal nutrition: bridging innovation, health, and sustainability. Front Vet Sci. (2025) 12:1727126. doi: 10.3389/fvets.2025.1727126, 41487484 PMC12756971

[33] Elghandour MMY Tan ZL Abu Hafsa SH Adegbeye MJ Greiner R Ugbogu EA . *Saccharomyces cerevisiae* as a probiotic feed additive to non and pseudo-ruminant feeding: a review. J Appl Microbiol. (2020) 128:658–74. doi: 10.1111/jam.14416, 31429174

[34] Sumanu VO Naidoo V Oosthuizen M Chamunorwa JP. A technical report on the potential effects of heat stress on antioxidant enzymes activities, performance and small intestinal morphology in broiler chickens administered probiotic and ascorbic acid during the hot summer season. Animals. (2023) 13:3407. doi: 10.3390/ani13213407 37958162 PMC10650450

[35] Manafi M Hedayati M Mirzaie S. Probiotic *Bacillus* species and *Saccharomyces boulardii* improve performance, gut histology and immunity in broiler chickens. S Afr J Anim Sci. (2018) 48:379–89. doi: 10.4314/sajas.v48i2.19

[36] Tinrat S Jiraprasertwong O. Probiotic potential and phytase-producing capacity of yeast isolated from Thai traditional fermentation starter (look-pang). Folia Microbiol. (2025) 70:1–17. doi: 10.1007/s12223-025-01314-z 40824521

[37] Thomas J. How Does Hindgut Fermentation Work in the Horse? Mad Barn (2023). p. 1–5.

[38] Cooke CG Gibb Z Harnett JE. The safety, tolerability and efficacy of probiotic bacteria for equine use. J Equine Vet Sci. (2021) 99:103407. doi: 10.1016/j.jevs.2021.103407, 33781424

[39] Ishizaka S Matsuda A Amagai Y Oida K Jang H Ueda Y . Oral administration of fermented probiotics improves the condition of feces in adult horses. J Equine Sci. (2014) 25:65–72. doi: 10.1294/jes.25.65, 25558179 PMC4266753

[40] Perricone V Sandrini S Irshad N Comi M Lecchi C Savoini G . The role of yeast *Saccharomyces cerevisiae* in supporting gut health in horses: an updated review on its effects on digestibility and intestinal and fecal microbiota. Animals. (2022) 12:3475. doi: 10.3390/ani12243475, 36552396 PMC9774806

[41] Cao Y Fan Z Jiang L Zhou Y Zhu B Xu J . Systematic evaluation and strategic utilization of bioactive constituents in solid-state fermentation-derived yeast culture. Syst Microbiol Biomanuf. (2026) 6:23. doi: 10.1007/s43393-025-00418-4

[42] Ganda E Chakrabarti A Sardi MI Tench M Kozlowicz BK Norton SA . *Saccharomyces cerevisiae* fermentation product improves robustness of equine gut microbiome upon stress. Front Vet Sci. (2023) 10:1134092. doi: 10.3389/fvets.2023.1134092, 36908513 PMC9998945

[43] Mirzaei M Mollakhalili-meybodi N Akrami Mohajeri F Shamsi F Khalili Sadrabad E. Technological characteristics of wheat germ flour fermented by paraprobiotic strains of *Lactobacillus acidophilus* and *Lactiplanti Bacillus plantarum*. J Food Meas Charact. (2026):1–9.

[44] Żak-Bochenek A Żebrowska-Różańska P Bajzert J Łaczmański Ł Szponar B Siwińska N . Investigating the potential immunomodulatory effects of commercial oral probiotic supplements on equine gastrointestinal tract barrier function. Front Immunol. (2025) 15:1487664. doi: 10.3389/fimmu.2024.1487664, 39906737 PMC11790434

[45] Borovkov S Kolchyk O Paliy A Borovkova V Zlenko O Pavlichenko O. Effect of probiotic complex of spore-forming bacteria Bacillus on the intestinal microbiome of normal and overweight horses. Croat Vet J. (2026) 57:94–103.

[46] Sun J Gu X Zhang H Zhao L Wang J Wang X . Application of probiotics in cats and dogs: benefits and mechanisms. Vet Sci. (2025) 12:1008. doi: 10.3390/vetsci12101008, 41150148 PMC12568152

[47] Rees P Salavati S. Evidence for the use of probiotics in small animals with chronic enteropathy. Companion Anim. (2026):2–7.

[48] Mârza SM Munteanu C Papuc I Radu L Purdoiu RC. The role of probiotics in enhancing animal health: mechanisms, benefits, and applications in livestock and companion animals. Animals. (2025) 15:2986. doi: 10.3390/ani15202986, 41153915 PMC12560942

[49] Zhang R Hu W Zhong S Chen W Xie S Chen M . The alleviating effects and mechanisms of *Enterococcus faecium* Kimate-X and *Lactobacillus plantarum* Kimate-F combination on canine inflammatory bowel disease. Front Vet Sci. (2025) 12:1534665. doi: 10.3389/fvets.2025.1534665, 40395807 PMC12090933

[50] Onuma M Ataka K Murakami A. Evaluating the safety and functionality of a novel compound containing prebiotics, probiotics, and postbiotics in healthy cats and dogs. Open Vet J. (2025) 15:1969–81. doi: 10.5455/OVJ.2025.v15.i5.11, 40557076 PMC12184448

[51] Dickerson SM Timlin CL Mccracken FB Skaggs P Nixon SL Day R . *Bifidobacterium animalis* subspecies *lactis* CECT 8145 affects markers of metabolic health in dogs during weight gain and weight loss. Animals. (2026) 16:259. doi: 10.3390/ani16020259, 41594449 PMC12837192

[52] Sivamaruthi BS Kesika P Chaiyasut C Fukngoen P Sisubalan N. A review of probiotic supplementation and its impact on the health and well-being of domestic cats. Vet Sci. (2025) 12:703. doi: 10.3390/vetsci12080703, 40872654 PMC12389776

[53] Pereira WA Franco SM Reis IL Mendonça CM Piazentin AC Azevedo PO . Beneficial effects of probiotics on the pig production cycle: an overview of clinical impacts and performance. Vet Microbiol. (2022) 269:109431. doi: 10.1016/j.vetmic.2022.109431, 35468401

[54] Taras D Vahjen W Simon O. Probiotics in pigs—modulation of their intestinal distribution and of their impact on health and performance. Livest Sci. (2007) 108:229–31. doi: 10.1016/j.livsci.2007.01.075

[55] Czech A Wlazło Ł Łukaszewicz M Lewińska A Nowakowicz-Dębek B. Comparative evaluation of *Bacillus subtilis* delivery forms reveals their effects on biogenic element excretion in pigs. Sci Rep. (2026). doi: 10.1038/s41598-026-41542-y PMC13103316 41813752

[56] Jonsson E Conway P. "Probiotics for pigs". In: Probiotics: The Scientific Basis. Dordrecht: Springer Netherlands (1992). p. 259–316.

[57] Ross GR Gusils C Oliszewski R De Holgado SC González SN. Effects of probiotic administration in swine. J Biosci Bioeng. (2010) 109:545–9. doi: 10.1016/j.jbiosc.2009.11.007 20471591

[58] Xie Q Yang M Feng L Chen J Yuan D Yin Y . Effect of *Lactobacillus reuteri* XY227 supplementation on meat quality, carcass characteristics and muscle fiber type in finishing pigs. Meat Sci. (2026) 234:110026. doi: 10.1016/j.meatsci.2025.110026, 41512799

[59] Zhao WH Liu HY Cai DM Kang DK Kim IH. Effects of multi-strain probiotic supplementation in low–crude protein diets on growth performance, apparent nutrient digestibility, Fecal microbial indicators, and nitrogen utilization in weaned piglets. Animals. (2026) 16:727. doi: 10.3390/ani16050727, 41828935 PMC12984969

[60] Adli DN Sjofjan O Sholikin MM Hidayat C Utama DT Jayanegara A . The effects of lactic acid bacteria and yeast as probiotics on the performance, blood parameters, nutrient digestibility, and carcase quality of rabbits: a meta-analysis. Ital J Anim Sci. (2023) 22:157–68. doi: 10.1080/1828051X.2023.2172467

[61] Colombino E Biasato I Michetti A Rubino MG Franciosa I Giribaldi M . Effects of dietary supplementation of *lactobacillus acidophilus* on blood parameters and gut health of rabbits. Animals. (2022) 12:3543. doi: 10.3390/ani12243543, 36552463 PMC9774759

[62] van der Sluis M Schreuder J de Greef KH. Improvement of Gastrointestinal Resilience in meat Rabbits: a Literature Review.Wageningen Livestock Research (2024).

[63] Khalifa WH Abo Sedera SA Abou-Hashim F. Reduction of postweaning stress by fasting regimen and probiotics supplementation and study its effects on cecal microbiota and physiological parameters of growing rabbits. J Anim Physiol Anim Nutr. (2024) 108:1588–94. doi: 10.1111/jpn.14000, 38879790

[64] Abdelsalam M Fathi M El-Raffa A Abd El-latif G Abou-Emera O Abd El-Fatah M . Influence of probiotic supplementation and rabbit line on growth performance, carcass yield, blood biochemistry and immune response under hot weather. Anim Biosci. (2025) 38:2033–42. doi: 10.5713/ab.24.0904, 40302681 PMC12415455

[65] Tufarelli V Losacco C Pugliese G Tateo A Schiavitto M Iarussi F . Effects of a multi-strain probiotic on productive traits, antioxidant defence, caecal microbiota and short-chain fatty acid profile, and intestinal histomorphology in rabbits. Anim Biosci. (2025) 38:1247–58. doi: 10.5713/ab.24.0716, 39901709 PMC12061584

[66] Mazziotta C Tognon M Martini F Torreggiani E Rotondo JC. Probiotics mechanism of action on immune cells and beneficial effects on human health. Cells. (2023) 12:184. doi: 10.3390/cells12010184, 36611977 PMC9818925

[67] Valeriano VDV Balolong MP Kang DK. Probiotic roles of *Lactobacillus* spp. in swine: insights from gut microbiota. J Appl Microbiol. (2017) 122:554–67. doi: 10.1111/jam.13364, 27914202

[68] Kritas SK. "Probiotics and prebiotics for the health of pigs and horses". In: Probiotics and Prebiotics in Animal Health and Food Safety. Cham: Springer (2018). p. 109–26.

[69] Sumanu VO Aluwong T Ayo JO Ogbuagu NE. Evaluation of changes in tonic immobility, vigilance, malondialdehyde and superoxide dismutase in broiler chickens administered fisetin and *Saccharomyces cerevisiae* and exposed to heat stress. J Vet Behav. (2019) 31:36–42.

[70] Lv W Ma Y Zhang Y Wang T Huang J He S . Effects of *Lactobacillus plantarum* fermented Shenling Baizhu San on gut microbiota, antioxidant capacity, and intestinal barrier function of yellow-plumed broilers. Front Vet Sci. (2023) 10:1103023. doi: 10.3389/fvets.2023.1103023, 36908522 PMC9992544

[71] Sandikci M Eren U Onol AG Kum S. Effects of heat stress and dietary *Saccharomyces cerevisiae* or bacitracin zinc on intestinal mucosa in quails. Rev Med Vet. (2004) 155:552–6.

[72] Mitin H Zulkifli I Che Jamri MH Zamzuri NA Samian NA Hussein AN . Alleviation of catching and crating stress by dietary supplementation of *Bacillus subtilis* in Pekin ducks. Animals. (2022) 12:3479. doi: 10.3390/ani12243479, 36552400 PMC9774105

[73] Ozen M Dinleyici EC. The history of probiotics: the untold story. Benef Microbes. (2015) 6:159–65. doi: 10.3920/BM2014.0103, 25576593

[74] Nawaz AH Zhang L. Oxidative stress in broiler chicken and its consequences on meat quality. Int J Life Sci Res Arch. (2021) 1:45–54.

[75] Broom LJ Kogut MH. Gut immunity: its development and reasons and opportunities for modulation in monogastric production animals. Anim Health Res Rev. (2018) 19:46–52. doi: 10.1017/S1466252318000026 29704909

[76] Williams NT. Probiotics. Am J Health Syst Pharm. (2010) 67:449–58. doi: 10.2146/ajhp090168, 20208051

[77] Nallala V Sadishkumar V Jeevaratnam K. Molecular characterization of antimicrobial *Lactobacillus* isolates and evaluation of their probiotic characteristics in vitro for use in poultry. Food Biotechnol. (2017) 31:20–41. doi: 10.1080/08905436.2016.1269289

[78] Anadon A Ares I Martinez-Larranaga MR Martinez MA. "Prebiotics and probiotics in feed and animal health". In: Nutraceuticals in Veterinary Medicine. Cham: Springer (2019). p. 261–85.

[79] Wang L Wang C Peng Y Zhang Y Liu Y Liu Y . Research progress on anti-stress nutrition strategies in swine. Anim Nutr. (2023) 13:342–60. doi: 10.1016/j.aninu.2023.03.006, 37214213 PMC10192683

[80] Mingmongkolchai S Panbangred W. *Bacillus* probiotics: an alternative to antibiotics for livestock production. J Appl Microbiol. (2018) 124:1334–46. doi: 10.1111/jam.13690, 29316021

[81] Yadav M Mandeep J Shukla P. Probiotics of diverse origin and their therapeutic applications: a review. J Am Coll Nutr. (2020) 39:469–79. doi: 10.1080/07315724.2019.1691957, 31765283

[82] Ezema C Ugwu CC. Yeast (*Saccharomyces cerevisiae*) as a probiotic of choice for broiler production. Benef Microorg Agric Aquac Areas. (2015) 13:59–79.

[83] Duan H Lu L Zhang L Li J Gu X Li J. Effects of *Lactobacillus lactis* supplementation on growth performance, hematological parameters, meat quality and intestinal flora in growing-finishing pigs. Animals. (2023) 13:1247. doi: 10.3390/ani13071247, 37048503 PMC10093238

[84] Lambo MT Chang X Liu D. The recent trend in the use of multistrain probiotics in livestock production: an overview. Animals. (2021) 11:2805. doi: 10.3390/ani11102805, 34679827 PMC8532664

[85] Lee J Park I Choi Y Cho J. *Bacillus* strains as feed additives: in vitro evaluation of their potential probiotic properties. Rev Colomb Cienc Pecu. (2012) 25:577–85.

[86] Kwoji ID Aiyegoro OA Okpeku M Adeleke MA. Multi-strain probiotics: synergy among isolates enhances biological activities. Biology. (2021) 10:322. doi: 10.3390/biology10040322, 33924344 PMC8070017

[87] Syngai GG Gopi R Bharali R Dey S Lakshmanan GA Ahmed G. Probiotics: the versatile functional food ingredients. J Food Sci Technol. (2016) 53:921–33. doi: 10.1007/s13197-015-2011-0 27162372 PMC4837740

[88] Gueimonde M Ouwehand AC Salminen S. Safety of probiotics. Scand J Nutr. (2004) 48:42–8. doi: 10.1080/11026480410026447

[89] Attia YA. Health benefits of probiotics in human and animal nutrition and prospective research. EC Nutr. (2019) 14:573–6.

[90] Biagi G. Pinna C., (2013). The utilization of probiotic bacterial strains for monogastric animals within the European Union. In: Proceeding of the 10th International Symposium Modern Trends in Livestock Production. Institute for Animal Husbandry, Belgrade, pp. 212–221.

[91] Pancheniak E Soccol CR. Isolation, Selection, Biochemical Characterization and Evaluation of Probiotic Potential of *Lactobacillus reuteri* LPB P01-001 in Swine, PhD Thesis. Curitiba, Brazil: Federal University of Paraná (2005). p. 78–89.

[92] Ahmed M Ismail ZSH Elwardany I Abdel-Wareth AAA. In ovo feeding technique of probiotics in broiler chickens: achievements, prospective and challenges. SVU-Int J Agric Sci. (2023) 5:95–126. doi: 10.21608/svuijas.2023.218144.1293

[93] Grandmont A Rhouma M Letourneau-Montminy MP Theriault W Mainville I Arcand Y . Characterization of the effects of a novel probiotic on *Salmonella* colonization of a piglet-derived intestinal microbiota using an improved bioreactor. Animals. (2024) 14:787. doi: 10.3390/ani14050787 38473172 PMC10930937

[94] Sumanu VO Aluwong T Ayo JO Ogbuagu NE. Cloacal temperature responses of broiler chickens administered fisetin and *Saccharomyces cerevisiae* and exposed to heat stress. Exp Results. (2021) 2:e24

[95] Alloui MN Szczurek W Swiatkiewicz S. The usefulness of prebiotics and probiotics in modern poultry nutrition: a review. Ann Anim Sci. (2013) 13:17–32.

[96] Asml AAA Invernizzi G Bontempo V Savoini G. The beneficial role of probiotics in monogastric animal nutrition and health. J Dairy Vet Anim Res. (2015) 2:00041

[97] Bortoluzzi C Barbosa JGM Pereira R Fagundes NS Rafael JM Menten JFM. Autolyzed yeast (*Saccharomyces cerevisiae*) supplementation improves performance while modulating the intestinal immune system and microbiology of broiler chickens. Front Sustain Food Syst. (2018) 2:85. doi: 10.3389/fsufs.2018.00085

[98] Salahi A Abd El-Ghany WA. Beyond probiotics: uses of next-generation probiotics for poultry and humans. J Anim Physiol Anim Nutr. (2024) 108:1336–47. doi: 10.1111/jpn.13972 38689488

[99] Santini C Baffoni L Gaggia F Granata M Gasbarri R Di Gioia D . Characterization of probiotic strains as feed additives in poultry against *Campylobacter jejuni*. Int J Food Microbiol. (2010) 141:S98–S108. doi: 10.1016/j.ijfoodmicro.2010.03.039 20452074

[100] Yoruk MA Gul M Hayirli A Macit M. Effects of humate and probiotic supplementation on egg production and quality during late laying period in hens. Poult Sci. (2004) 83:84–8. doi: 10.1093/ps/83.1.84, 14761088

[101] Wang WC Yan FF Hu JY Amen OA Cheng HW. Supplementation of *Bacillus subtilis*-based probiotic reduces heat stress-related behaviors and inflammatory response in broiler chickens. J Anim Sci. (2018) 96:1654–66. doi: 10.1093/jas/sky092, 29528406 PMC6140875

[102] Bai SP Wu AM Ding XM Lei Y Bai J Zhang KY . Effects of probiotic-supplemented diets on growth performance and intestinal immune characteristics of broiler chickens. Poult Sci. (2013) 92:663–70. doi: 10.3382/ps.2012-02813, 23436517

[103] Liao SF Nyachoti M. Using probiotics to improve swine gut health and nutrient utilization. Anim Nutr. (2017) 3:331–43. doi: 10.1016/j.aninu.2017.06.007, 29767089 PMC5941265

[104] Samolinska W Kowalczuk-Vasilev E Grela ER. Comparative effect of different dietary inulin sources and probiotics on growth performance and blood characteristics in growing-finishing pigs. Arch Anim Nutr. (2018) 72:379–95. doi: 10.1080/1745039X.2018.1505147 30183392

[105] Swain BK Naik PK Chakurkar EB Singh NP. Effect of combined supplementation of probiotic and yeast on growth, carcass characteristics and economics of broiler production. Anim Nutr Feed Technol. (2012) 12:103–10.

[106] Alkhalf A Alhaj M Al-Homidan I. Influence of probiotic supplementation on blood parameters and growth performance in broiler chickens. Saudi J Biol Sci. (2010) 17:219–25. doi: 10.1016/j.sjbs.2010.04.005, 23961081 PMC3730717

[107] Naeem M Bourassa D. Probiotics in poultry: unlocking productivity through microbiome modulation and gut health. Microorganisms. (2025) 13:257. doi: 10.3390/microorganisms13020257, 40005624 PMC11857632

[108] Surai PF Ivan I Kochish VI Michael TK. Antioxidant defence systems and oxidative stress in poultry biology: an update. Antioxidants. (2019) 8:235. doi: 10.3390/antiox8070235 31336672 PMC6680731

[109] Schmid K Schlothauer RC Friedrich U Staudt C Apajalahti J Hansen EB. "Development of probiotic food ingredients". In: Probiotics in Food Safety and Human Health. Boca Raton: CRC Press (2006). p. 35–66.

[110] Tamime AY. Fermented milks: a historical food with modern applications. Eur J Clin Nutr. (2002) 56:S2–S15. doi: 10.1038/sj.ejcn.1601657 12556941

[111] Sumanu VO Naidoo V Oosthuizen MC, Chamunorwa JP. Evaluating the efficacy of probiotics and ascorbic acid as anti-stress agents against heat stress in broiler chickens. Frontiers in Veterinary Science (2024) 11:1482134. 39502951 10.3389/fvets.2024.1482134 PMC11534807

[112] Vieco-Saiz N Belguesmia Y Raspoet R Auclair E Gancel F Kempf I . Benefits and inputs from lactic acid bacteria and their bacteriocins as alternatives to antibiotic growth promoters during food-animal production. Frontiers in microbiology (2019) 10:57. 30804896 10.3389/fmicb.2019.00057 PMC6378274

[113] Gueimonde M Corzo N Vinderola G Reinheimer J de los Reyes-Gavilan CG. Evolution of carbohydrate fraction in carbonated fermented milks as affected by β-galactosidase activity of starter strains. J*ournal of dairy research* (2002) 69, 125–137. 10.1017/s0022029901005192 12047103

[114] De La Guardia-Hidrogo VM Soto-Diaz K Rummell LM Valizadegan N Fields CJ. Steelman AJ, et al Effects of yeast-enriched functionalized canola meal supplementation on apparent total tract macronutrient digestibility and fecal characteristics, fecal microbiota, and immune function of healthy adult dogs. Journal of animal science (2024) 102:skae224. 39101402 10.1093/jas/skae224 PMC11350369

[115] Poorni S Srinivasan MR, Nivedhitha MS. Probiotic Streptococcus strains in caries prevention: A systematic review. Journal of Conservative Dentistry and Endodontics (2019) 22, 123–128. 10.4103/JCD.JCD_505_18 PMC6519182 31142979

